# Prognostic role of pre-treatment C-reactive protein/albumin ratio in esophageal cancer: a meta-analysis

**DOI:** 10.1186/s12885-019-6373-y

**Published:** 2019-11-29

**Authors:** Zhenhua Liu, Hongtai Shi, Longyun Chen

**Affiliations:** 1 Department of Radiation Oncology, Yancheng No.1 People’s Hospital, 66 Renmin Road, Yancheng, 224000 China; 2 Department of Radiation Oncology, The Third People’s Hospital of Yancheng, 75 Juchang Street, Yancheng, 224005 China

**Keywords:** Esophageal cancer, C-reactive protein/albumin ratio, Prognosis, Meta-analysis

## Abstract

**Background:**

In recent years, the role of pre-treatment C-reactive protein/albumin ratio (CAR) in prognosis of esophageal cancer (EC) has been investigated by several studies. This meta-analysis aimed to provide a more accurate and objective assessment of the prognostic value of pre-treatment CAR in EC.

**Methods:**

Studies assessing the role of pre-treatment CAR in prognosis of EC were searched from PubMed, Embase and the Cochrane Library (last update by April 16, 2019). The hazard ratios (HRs) of CAR and the corresponding 95% CIs for overall survival (OS) or cancer-specific survival (CSS) in EC were extracted for pooled analysis.

**Results:**

A total of eight observational studies including 2255 patients were collected. The pooled analysis showed that high CAR was related to worse OS in EC (pooled HR = 1.81; 95% CI = 1.40–2.35; *P* < 0.001). Subgroup analyses showed that the negative correlation between the CAR and OS was consistently demonstrated in subgroups stratified by country, pathological type, and cut-off value (*P* < 0.05). However, there was no relation between CAR and OS in subgroup of patients receiving neoadjuvant chemotherapy at a proportion of 100% (HR = 1.15, 95% CI = 0.56–2.69; *P* = 0.715). In addition, high CAR was also related to worse CSS in EC (pooled HR = 2.61; 95% CI = 1.67–4.06; *P* < 0.001).

**Conclusions:**

High pre-treatment CAR was an adverse prognostic factor for EC patients. More large-sample clinical trials are still needed to verify the prognostic value of pre-treatment CAR in EC.

## Background

Esophageal cancer (EC) is one of the most common malignant tumors of digestive system [1]. 62.9% of new cases of EC in the world come from China, and are mainly esophageal squamous cell carcinoma (ESCC) [2, 3]. In addition, about 400,000 people die of EC every year in the world, and China’s deaths from EC accounts for about half of the world’s total deaths [2, 3]. Early diagnosis and correct treatment strategy based on prognostic assessment are the key to improve the prognosis of EC. Some biomarkers with high sensitivity and specificity are of great significance for prognostic assessment and determining the optimal treatment strategy. Currently, there is still a need for more research on prognostic biomarkers in EC, because few biomarkers have sufficient specificity and sensitivity to assess the prognosis of EC.

C-reactive protein (CRP), as an acute phase reactant (APR), is mainly produced by liver cells and regulated by interleukin-6 (IL-6, [4]. Albumin (Alb) can reflect the nutritional status of the body [5]. CRP/Alb ratio (CAR) is an indicator that reflects both inflammatory and nutritional status. Most patients with malignant tumors have elevated CAR [6, 7]. Elevated CAR indicates an increase in serum CRP concentration and hypoalbuminemia, suggesting that the overall condition of the patient is poor [8]. Studies have shown that CAR is related to prognosis in different tumors [9–11].

The relation between CAR and the prognosis of EC has been investigated in several studies. For example, Ishibashi et al. [12] and Kunizaki et al. [13] reported that EC patients with a high pre-treatment CAR level had poor overall survival (OS) and cancer-specific survival (CSS). However, another study showed a different result that the prognosis of EC patients was not related to the pre-treatment CAR level [14]. Because the role of CAR in the prognosis of EC is still controversial, we meta-analyzed several independent studies investigating the prognostic role of CAR in EC.

## Methods

### Search strategy

For this meta-analysis, the PRISMA guidelines were followed [15]. PubMed, Embase and the Cochrane Library (last update by April 16, 2019) were used to search the literatures. C-Reactive Protein, Albumins and Esophageal Neoplasms were the Medical Subject Heading (MeSH) terms. The language was not restricted. The text of all retrieved literatures was read, and the reference lists were also searched to supply data.

### Inclusion criteria

The included studies must meet the following criteria: (1) Studies investigated the role of CAR in the prognosis of EC; (2) Serum CRP and albumin levels were obtained prior to operation and chemotherapy; (3) The hazard ratio (HR) and 95% confidence intervals (CI) for survival were reported or could be figured out.

### Data extraction

The first author’s surname, publication year, country, case number, pathological type, proportion of patients receiving neoadjuvant chemotherapy (NAC), tumor location, Tumor Node Metastasis (TNM) stage, length of follow-up, cut-off value, analysis method, HRs of CAR for survival and the corresponding 95% CIs were extracted. Compared with the results of univariate analyses, the results of multivariate analyses were prioritized because the latter adjusted the confounding factors.

### Quality assessment

The quality of each included study was evaluated using the Newcastle-Ottawa Quality Assessment Scale (NOS). The score of NOS ranges from 0 to 9. A score above 5 represents high quality.

### Statistical analysis

All enrolled studies defined high and low CARs using the optimal cut-off value. The prognostic value of CAR in EC patients was assessed by HR and 95% CI. When the HR and 95% CI was not directly reported, we figured out them from Kaplan-Meier survival curves using the method of Tierney [16]. When necessary, we e-mailed the corresponding author to get additional information or original data. Chi-square test and the *I*^2^ statistic were used to assess the heterogeneity [17, 18]. When there was significant heterogeneity (*P* value of chi-square test < 0.05 and/or *I*^2^ ≥ 25%), the random-effects model was used [19], otherwise the fixed-effects model was used [20]. Begg’s and Egger’s tests and funnel plot were used to assess the publication bias. When publication bias was observed (*P* values of Begg’s and Egger’s tests < 0.05 and/or the funnel plot was asymmetric), the “Trim and Fill” method was used to adjust the bias [21]. All analyses and figures were performed or generated using STATA version 12.0. A *P* value less than 0.05 represented statistical significance.

## Results

### Study characteristics

**Fig. 1:**
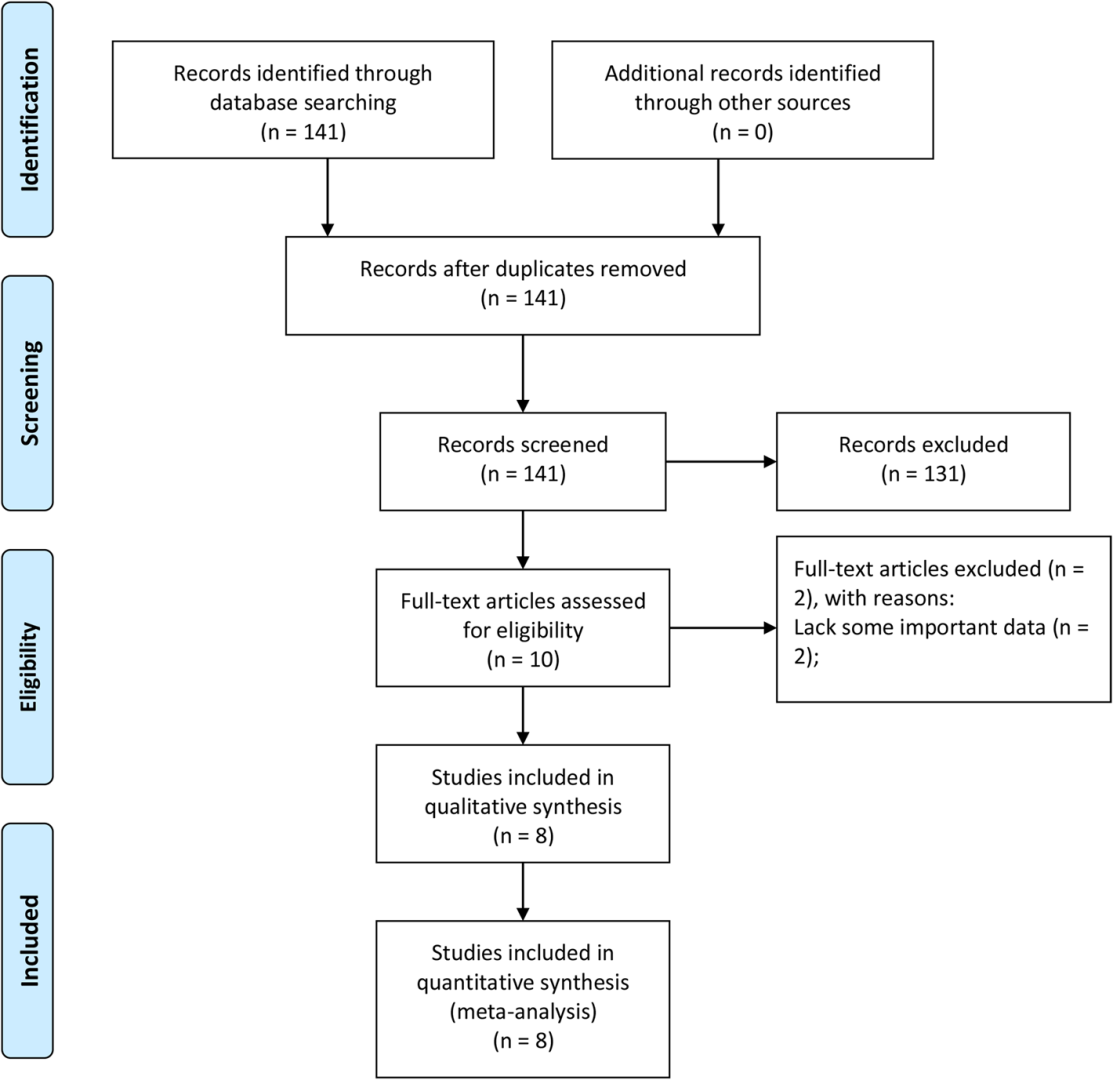
Flow diagram of the study selection process

By retrieving the database, we initially identified 141 studies. After the preliminary screening, 131 studies were excluded because they did not investigate the role of CAR in the prognosis of EC, and then the remaining ten studies were assessed by reading the full text. Among these, two studies without some important data were excluded. Finally, eight studies with 2255 patients were included (Fig. 1) [12–14, 22–26]. Their mean score of NOS was 7 (range 5–9) (Table 1).

**Table 1:** Main characteristics of all studies included in the meta-analysis

Study	Country	Case number	High expression (%)	Pathological type	NAC (%)	Location	Tumor stage	Follow-up (months)	Cut-off value	Multivariate analysis	HRs provided from	Outcome	NOS score
Ishibashi 2018 [12]	Japan	143	75 (51.7)	Multiple	72 (50.3)	22/56/65	33/33/60/17	NR	0.085	Yes/No	Report/SC	OS/CSS	7
Kunizaki 2018 [13]	Japan	116	51 (44)	Squamous	NR	15/31/19	0-II36/III-IV29	Median 35.9	0.042	Yes/No	Report/SC	OS/CSS	7
Otowa 2017 [14]	Japan	149	114 (76.5)	Squamous	149 (100)	24/73/52	IIA24/IIB36/III89	NR	0.03	Yes	Report	OS	6
Shao 2015 [24]	China	633	140 (22.1)	Squamous	0 (0)	33/428/172	I106/IIa203/IIb92/III232	Median 39	0.012	Yes	Report	OS	7
Matsunaga 2019 [25]	Japan	163	69 (42.3)	Multiple	95 (58.3)	32/86/45	012/I48/II45/III54/IV4	NR	0.0375	No	SC	OS	7
Wei 2015 [22]	China	423	147 (34.8)	Squamous	NR	36/252/135	41/131/81/23	Median 35.7	0.055	Yes	Report	OS	8
Xu 2015 [23]	China	468	87 (18.6)	Squamous	0 (0)	155/64/249	I24/II181/IIIA121/IIIB+IIIC142	Median 49.9	0.5	Yes	Report	OS	9
Yu 2018 [26]	China	160	85 (53.1)	Squamous	0 (0)	26/104/30	I160	Median 71.8	0.023	Yes	Report	OS	5

*NAC* neoadjuvant chemotherapy, *NR* not report, *HR* hazard ratio, *SC* survival curve, *OS* overall survival, *CSS* cancer-specific survival, *NOS* Newcastle-Ottawa Quality Assessment Scale

The main characteristics of included studies were showed in Table 1. The number of patients in each study ranged from 116 to 633. Patients came from two countries: China or Japan. Six studies included only ESCC, while other two studies included multiple pathological types of EC. In three studies, the proportion of patients receiving NAC was 0%, and in one study it was 100%. EC in each study was distributed in the upper, middle and lower segments of esophagus. All studies used the optimal cut-off values. Only two studies focused on patients’ CSS. HRs for OS were reported directly in seven studies. HRs for CCS in two studies were both estimated indirectly.

### Overall survival

**Fig. 2:**
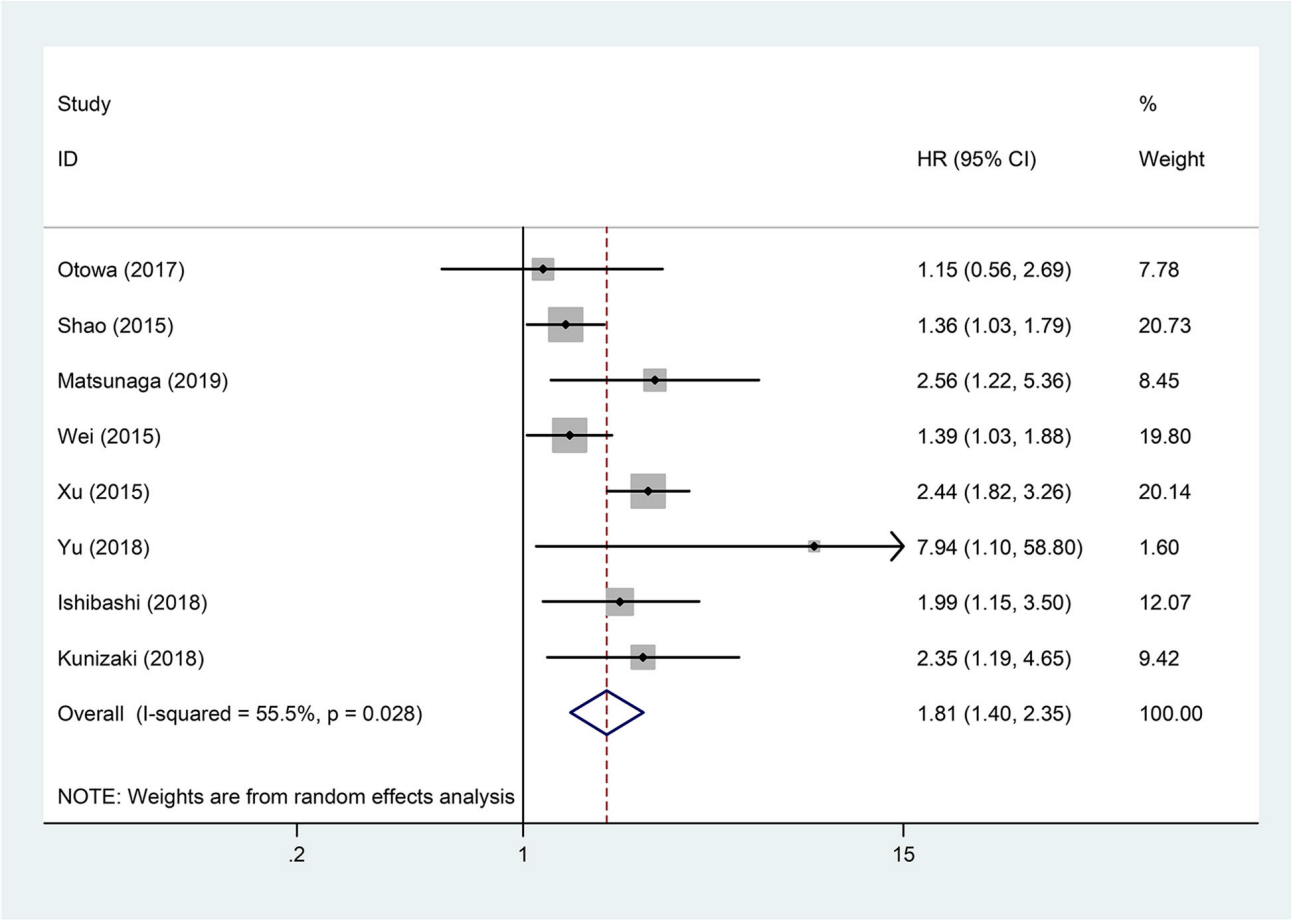
Forest plot of studies evaluating the hazard ratio of high C-reactive protein/albumin ratio for overall survival of esophageal cancer patients

Table 2 showed the main results of pooled analyses. Eight articles with 2255 patients investigated the influence of CAR to OS in EC. Because there was significant heterogeneity among these studies (*I*^2^ = 55.5%, *P* = 0.028), a random-effects model was used for the pooled analysis. The result showed that high CAR was related to worse OS (pooled HR = 1.81; 95% CI = 1.40–2.35; *P* < 0.001) (Fig. 2). We further conducted subgroup analyses, sensitivity analysis and meta-regression to explore the sources of heterogeneity.

**Table 2:** Pooled hazard ratios for OS and CSS according to subgroup analyses

Outcome subgroup	Study number	Case number	HR (95%CI)-model	*P* value	Heterogeneity	
					*I*^2^ (%)	*P*
OS	8	2255	1.81 (1.40–2.35)-random	< 0.001	55.5	0.028
Country						
Japan	4	571	1.98 (1.41–2.77)-fixed	< 0.001	0	0.469
China	4	1684	1.76 (1.19–2.59)-random	0.005	75.9	0.006
Pathological type						
Squamous	6	1949	1.73 (1.26–2.38)-random	0.001	65.0	0.014
Multiple	2	306	2.18 (1.40–3.40)-fixed	0.001	0	0.597
Proportion of NAC						
100%	1	149	1.15 (0.56–2.69)	0.715		
None	3	1261	2.02 (1.13–3.62)-random	< 0.001	80.7	0.006
Others	4	845	1.79 (1.32–2.41)-random	< 0.001	25.5	0.259
Cut-off value						
< 0.04	4	1105	1.68 (1.03–2.73)-random	0.037	46.6	0.132
≥ 0.04	4	1150	1.94 (1.41–2.69)-random	< 0.001	59.0	0.063
CSS	2	259	2.61 (1.67–4.06)-fixed	< 0.001	0	0.748

*OS* overall survival, *CSS* cancer-specific survival, *NAC* neoadjuvant chemotherapy, *HR* hazard ratio, *CI* confidence interval

**Fig. 3:**
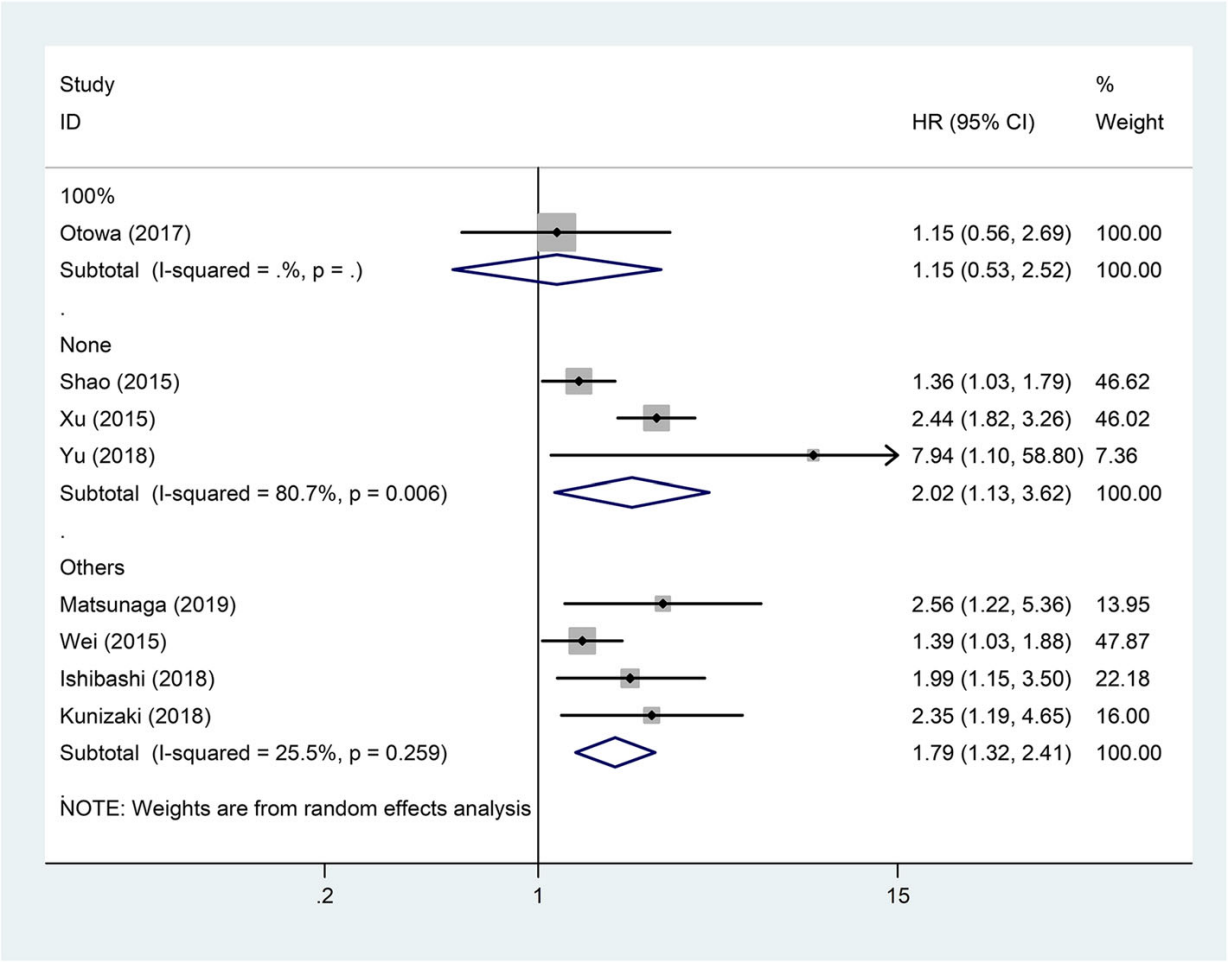
Forest plot of studies evaluating the hazard ratio of high C-reactive protein/albumin ratio for overall survival of esophageal cancer patients divided by the ratio of neoadjuvant chemotherapy

Subgroup analyses showed that the negative correlation between CAR and OS was consistently demonstrated in subgroups stratified by country, pathological type, and cut-off value (*P* < 0.05, Table 2). The relationship between CAR and OS was only affected by the proportion of patients receiving NAC. In subgroup of patients receiving NAC at a proportion of 100%, there was no relation between CAR and OS (HR = 1.15, 95% CI = 0.56–2.69; *P* = 0.715) (Fig. 3).

**Fig. 4:**
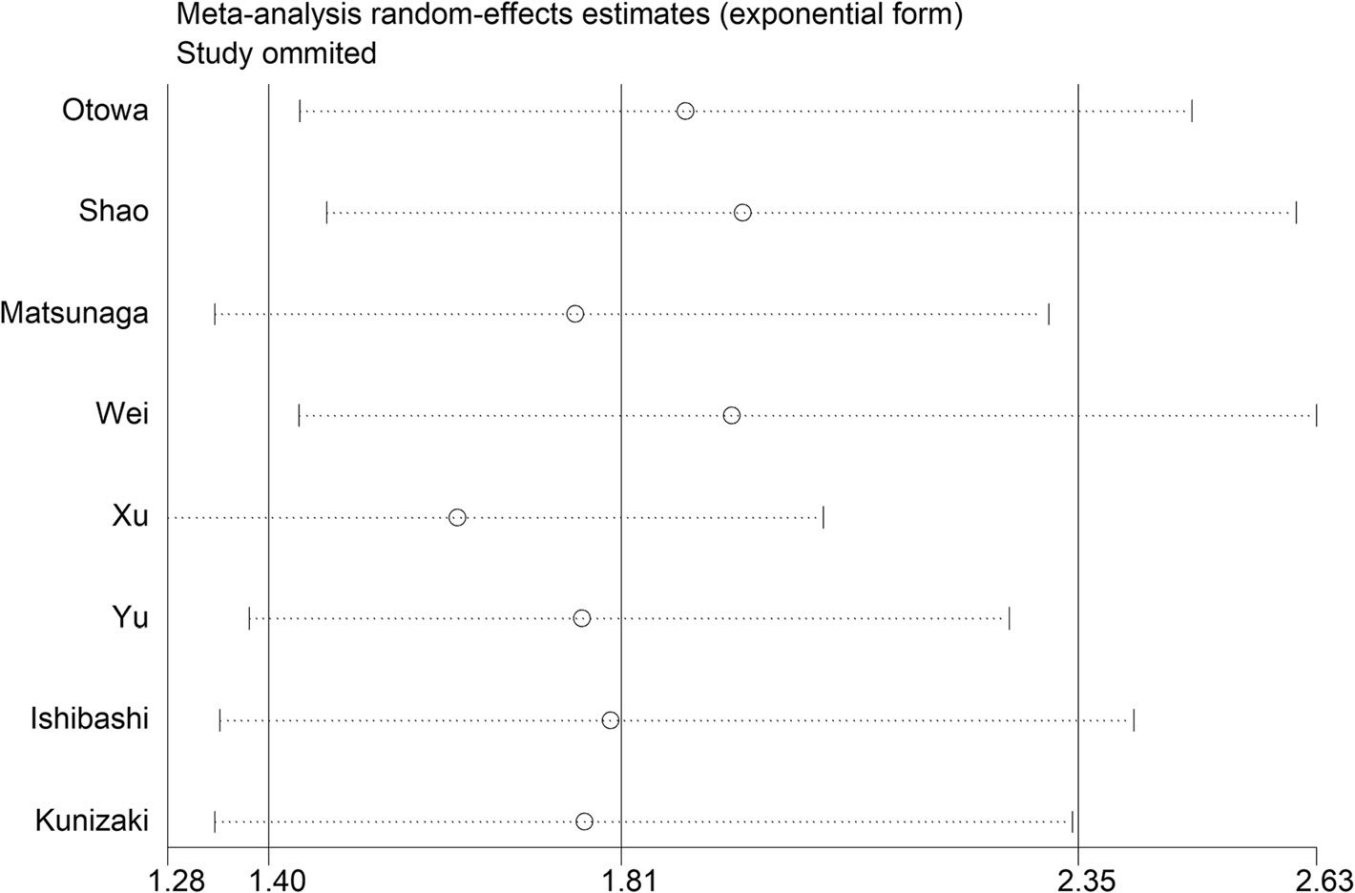
Sensitivity analysis of studies evaluating the relationship between C-reactive protein/albumin ratio and patients’ overall survival in esophageal cancer

Sensitivity analysis showed that deletion of any study did not change the negative association between CAR and OS in EC (Fig. 4). Meta-regression showed that the pooled effect size was not significantly impacted by the country (*P* = 0.674), NAC rate (*P* = 0.585), pathological type (*P* = 0.481), and cut-off value (*P* = 0.518).

**Fig. 5:**
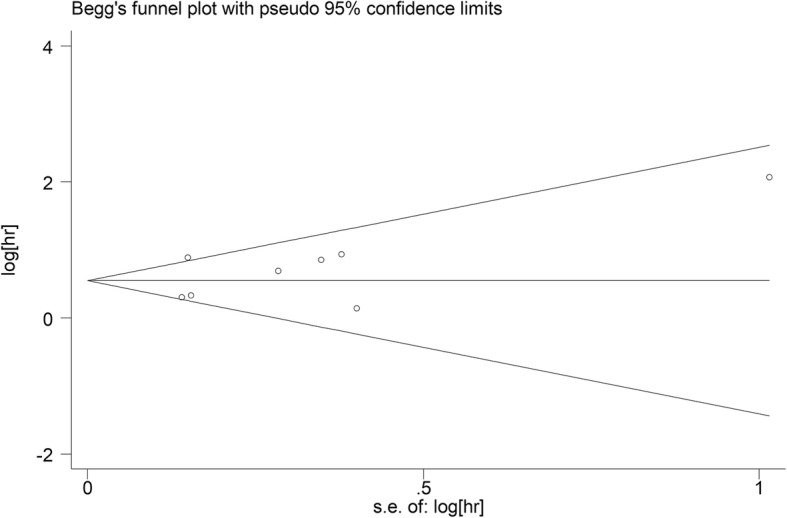
Funnel plot of publication bias for studies evaluating the relationship between C-reactive protein/albumin ratio and patients’ overall survival in esophageal cancer

Because the funnel plot was asymmetrical (Fig. 5), a publication bias existed among these studies even though the *P* values of Egger’s and Begg’s tests were both greater than 0.05. The “Trim and Fill” method was used to adjust the publication bias. After the adjustment, the pooled HR of CAR for OS in EC was 1.71 (95% CI = 1.33–2.21; *P* < 0.001).

### Cancer-specific survival

**Fig. 6:**
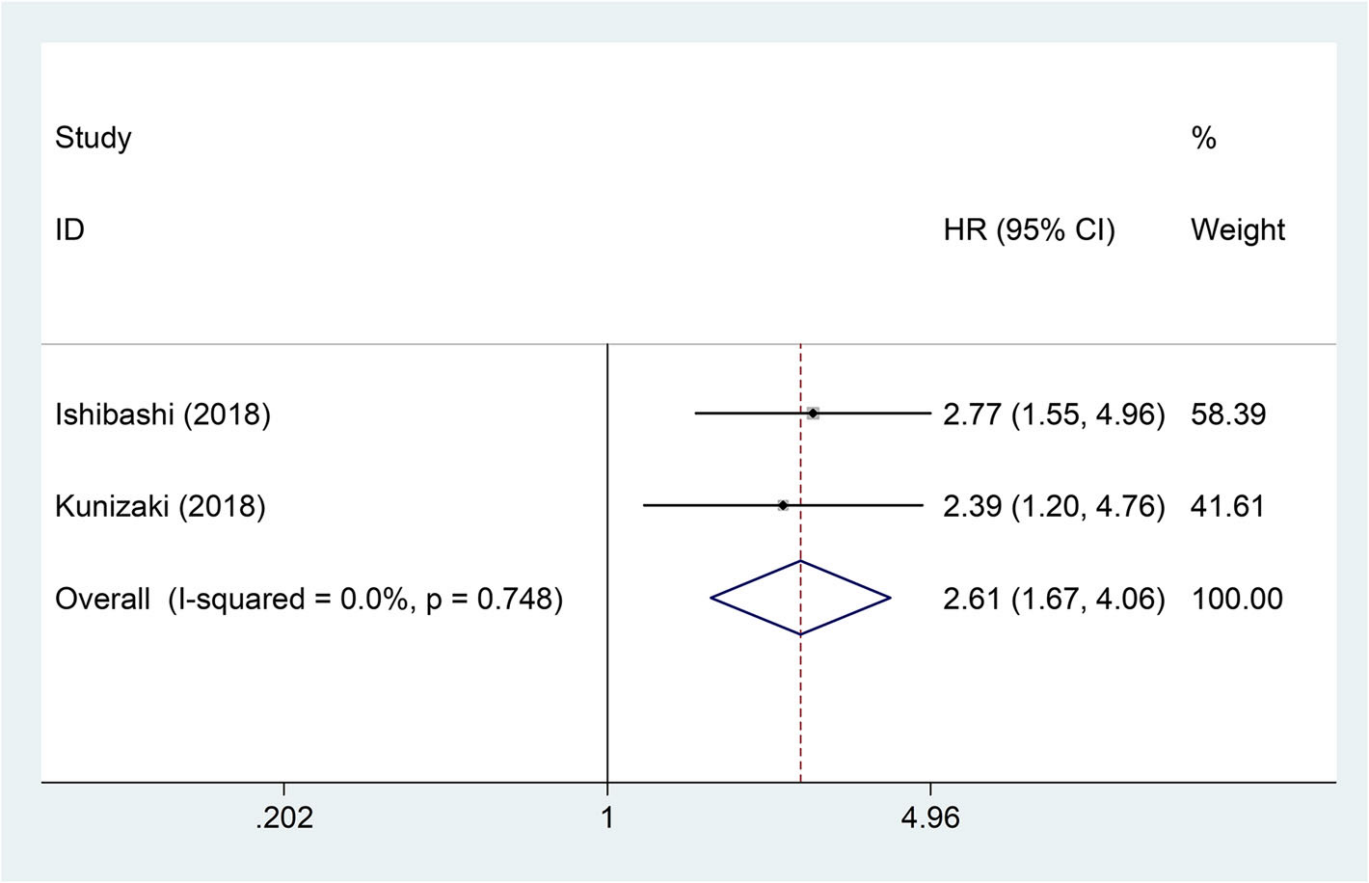
Forest plot of studies evaluating the hazard ratio of high C-reactive protein/albumin ratio for cancer-specific survival of esophageal cancer patients

Two studies with 259 patients investigated the influence of CAR to CSS in EC. Because there was no heterogeneity among these studies (*I*^2^ = 0%, *P* = 0.748), a fixed-effects model was used. The pooled analysis showed that high CAR was related to worse CSS in EC (pooled HR = 2.61; 95% CI = 1.67–4.06; *P* < 0.001) (Table 2; Fig. 6).

## Discussion

Recently, many studies have indicated that inflammation-based biological indicators for measuring the severity of systemic inflammatory reaction, such as CAR, Glasgow Prognostic Score (GPS), modified GPS (mGPS), neutrophil-to-lymphocyte ratio (NLR), and platelet-to-lymphocyte ratio (PLR), have prognostic value in various types of tumor, including EC [27–30]. Compared with other tumors, EC often causes difficulty in eating, so the proportion of malnutrition and cachexia in EC patients is relatively high. In addition, EC patients often receive multiple treatments such as chemotherapy, radiation therapy, and surgery. The inappetence and increased consumption caused by treatment will further aggravate the malnutrition. Low serum albumin level, as an indicator of malnutrition status, is associated with survival outcomes in EC [31, 32]. Therefore, CAR calculated from serum CRP and albumin concentrations is particularly suitable for assessing the prognosis of EC patients. In addition, CAR can influence the clinical decision-making of EC. Because patients with high CAR usually have severe tumor-related inflammatory reaction or poor nutritional status, these patients may benefit from anti-inflammatory therapy or nutritional support. In other words, anti-inflammatory therapy and nutritional support can be added to the individualized treatment regimen of EC patients with high CAR.

Besides CAR, GPS and mGPS are two other biological indicators based on serum CRP and albumin concentrations, which can also be used as independent prognostic factors in EC [24, 33]. GPS and mGPS are calculated by converting serum CRP and albumin concentrations into categorical variables, while CAR is directly calculated from numerical variables of serum CRP and albumin. Therefore, compared with GPS and mGPS, CAR is easier to apply. In addition, Shao et al. [24] found that most ESCC patients were allocated to the group of score 0 according to mGPS and GPS, but according to CAR, they can be evenly grouped. Therefore, CAR seems to have a wider clinical application than GPS and mGPS. Backward stepwise selection used in their study also demonstrated that the CAR, instead of GPS and mGPS, was chosen to build the best-fit prediction model. Furthermore, Liu et al. [34] also revealed that the CAR was superior to GPS and mGPS, because the CAR had a higher value of area under the curve (AUC).

The present meta-analysis, including cohort data of 2255 patients from 8 studies, provides strong evidence that a high pretreatment CAR is a predictor of poor survival for EC patients. Subgroup analyses showed that negative correlation between pretreatment CAR and OS was not affected by country, pathological type, or cut-off value. Only in subgroup of patients receiving NAC at a proportion of 100%, CAR had no influence to OS. In addition, this meta-analysis showed that high CAR was also related to worse CSS for EC patients.

This is the first meta-analysis focusing on the role of CAR in the prognosis of EC. However, some shortages of this meta-analysis should also be pointed out. First, this meta-analysis included only eight studies. The small number of included studies led to a lack of sufficient data to support the results of the subgroup analyses. For example, the result from the subgroup analysis based on NAC ratio showed that CAR was not associated with patients’ OS in subgroup of 100%, but this subgroup contained only one study, so the evidence may not be sufficient. Second, since the included studies were from China or Japan, ESCC was the most important type of EC in these two countries [35], so no studies have evaluated the role of CAR in the prognosis of esophageal adenocarcinoma alone. The subgroup analysis based on pathological type in this meta-analysis also did not contain adenocarcinoma subgroup. However, in North America and Europe, esophageal adenocarcinoma is the most important type of EC [35]. Therefore, our findings may be more applicable to EC patients in Asia than North America and Europe. Third, several HRs were from univariate analyses which may overestimate the effect size. Fourth, because the number of eligible studies was too small, a study with a NOS score of only 5 was also included in this meta-analysis, which reduced the quality of this meta-analysis to some extent. Finally, several HRs estimated from the survival curves may differ from the actual values.

## Conclusion

Pre-treatment CAR is a novel and promising inflammation-based prognostic indicator for EC patients. Due to its simplicity, utility and economy, pre-treatment CAR may be an important factor in improving clinical decision-making of EC. Of course, our results also need to be validated by large-sample clinical trials.

## Data Availability

Meta-analysis is a secondary analysis, which the data are all fully available without restriction, and all the material can be found in the included original studies.

## Abbreviations

APR: Acute phase reactant
CAR: C-reactive protein/albumin ratio
CI: Confidence intervals
CSS: Cancer-specific survival
EC: Esophageal cancer
ESCC: Esophageal squamous cell carcinoma
HR: Hazard ratio
IL-6: Interleukin-6
MeSH: Medical subject heading
NOS: Newcastle-Ottawa quality assessment scale
OS: Overall survival
TNM: Tumor node metastasis
